# Residential Dampness and Molds and the Risk of Developing Asthma: A Systematic Review and Meta-Analysis

**DOI:** 10.1371/journal.pone.0047526

**Published:** 2012-11-07

**Authors:** Reginald Quansah, Maritta S. Jaakkola, Timo T. Hugg, Sirpa A M. Heikkinen, Jouni J. K. Jaakkola

**Affiliations:** 1 Center for Environmental and Respiratory Health Research, University of Oulu, Oulu, Finland; 2 Respiratory Medicine Unit, Institute of Clinical Medicine, University of Oulu and Oulu University Hospital, Oulu, Finland; 3 Institute of Health Sciences, University of Oulu, Oulu, Finland; University of Bochum, Germany

## Abstract

**Context:**

Studies from different geographical regions have assessed the relations between indoor dampness and mold problems and the risk of asthma, but the evidence has been inconclusive.

**Objective:**

To assess the relations between indicators of indoor dampness and mold problems and the risk of developing new asthma, and to investigate whether such relations differ according to the type of exposure.

**Data sources:**

A systematic literature search of PubMed database from 1990 through March 2012 and the reference lists of recent reviews and of relevant articles identified in our search.

**Study selection:**

Cohort/longitudinal and incident case-control studies assessing the relation between mold/dampness and new asthma were included.

**Data extraction:**

Three authors independently evaluated eligible articles and extracted relevant information using a structured form.

**Synthesis:**

Sixteen studies were included: 11 cohort and 5 incident case-control studies. The summary effect estimates (EE) based on the highest and lowest estimates for the relation between any exposure and onset of asthma were 1.50 (95% confidence interval [CI] 1.25–1.80, random-effects model, *Q*-statistic 38.74 (16), *P* = 0.001) and 1.31 (95% CI 1.09–1.58, random-effects model, *Q*-statistic 40.08 (16), *P* = 0.000), respectively. The summary effect estimates were significantly elevated for dampness (fixed-effects model: EE 1.33, 95% CI 1.12–1.56, *Q*-statistic 8.22 (9), *P* = 0.413), visible mold (random-effects model; EE 1.29, 95% CI 1.04–1.60, 30.30 (12), *P* = 0.001), and mold odor (random-effects model; EE 1.73, 95% CI 1.19–2.50, *Q*-statistics 14.85 (8), *P* = 0.038), but not for water damage (fixed-effects model; EE 1.12, 95% CI 0.98–1.27). Heterogeneity was observed in the study-specific effect estimates.

**Conclusion:**

The evidence indicates that dampness and molds in the home are determinants of developing asthma. The association of the presence of visible mold and especially mold odor to the risk of asthma points towards mold-related causal agents.

## Introduction

Indoor dampness and mold problems are common around the world, being one of the most important indoor problems globally. In cold climate the prevalence of water damage and dampness problems has varied between 5% and 30%, while in moderate and warm climates the estimates have been between 10% and 60% [1]–[3]. The prevalence of indoor mold has been 5–10% in cold climate and 10–30% in moderate and warm climates [2], [3]. Thus, a substantial proportion of the world's population is exposed to dampness-related exposures.

Since the 1990s increasing number of studies from different geographical regions has addressed the health effects related to indoor dampness and mold problems. In 2004 the Institute of Medicine in the USA [4] reported a review of literature and concluded that there is evidence of an association between indoor dampness-related exposures and the following outcomes: upper respiratory tract symptoms, cough, wheeze, and asthma exacerbation. In addition, sufficient evidence was found between mold exposure and hypersensitivity pneumonitis. In a meta-analysis by Fisk and co-workers [5] the risk of current (prevalent) asthma and ever-diagnosed (prevalent) asthma were significantly increased in relation to any dampness or molds as a combined exposure, but the results on development of new asthma remained inconclusive, as only four studies had been published by then.

Asthma is the most common chronic disease in children and it is also a common chronic disease in adults with the prevalence of about 7% in Western countries [6], [7]. There has been debate on whether indoor dampness-related exposures can cause asthma, a question which is of major public health and economic importance. If development of asthma could be prevented by prevention or repairing of indoor dampness problems, this could lead to substantial savings in health care costs and improvement in public health. On the other hand, such remediation measures are often expensive, so clear evidence of chronic health effects would justify such investment in the indoor environments. A qualitative review up to late 2009 was published recently by Mendell et al [8], showing that there are new studies published on indoor molds and asthma development. However, they did not conduct any quantitative analysis of the studies.

To address whether studies published up till March 2012 support the hypothesis that indoor dampness and mold problems significantly increase the risk of developing new asthma and to provide a quantitative estimate for these relations, we carried out a systematic review and meta-analysis. In addition, we investigated whether such relations differ according to the type of exposure, by carrying out separate meta-analyses for different exposure indicators, including water damage, dampness, visible mold, and mold odor. Differences according to the type of exposure would give insight into the specific causal agents and pathways and be of importance for preventive actions.

## Materials and Methods

### Search strategy

**Figure 1:**
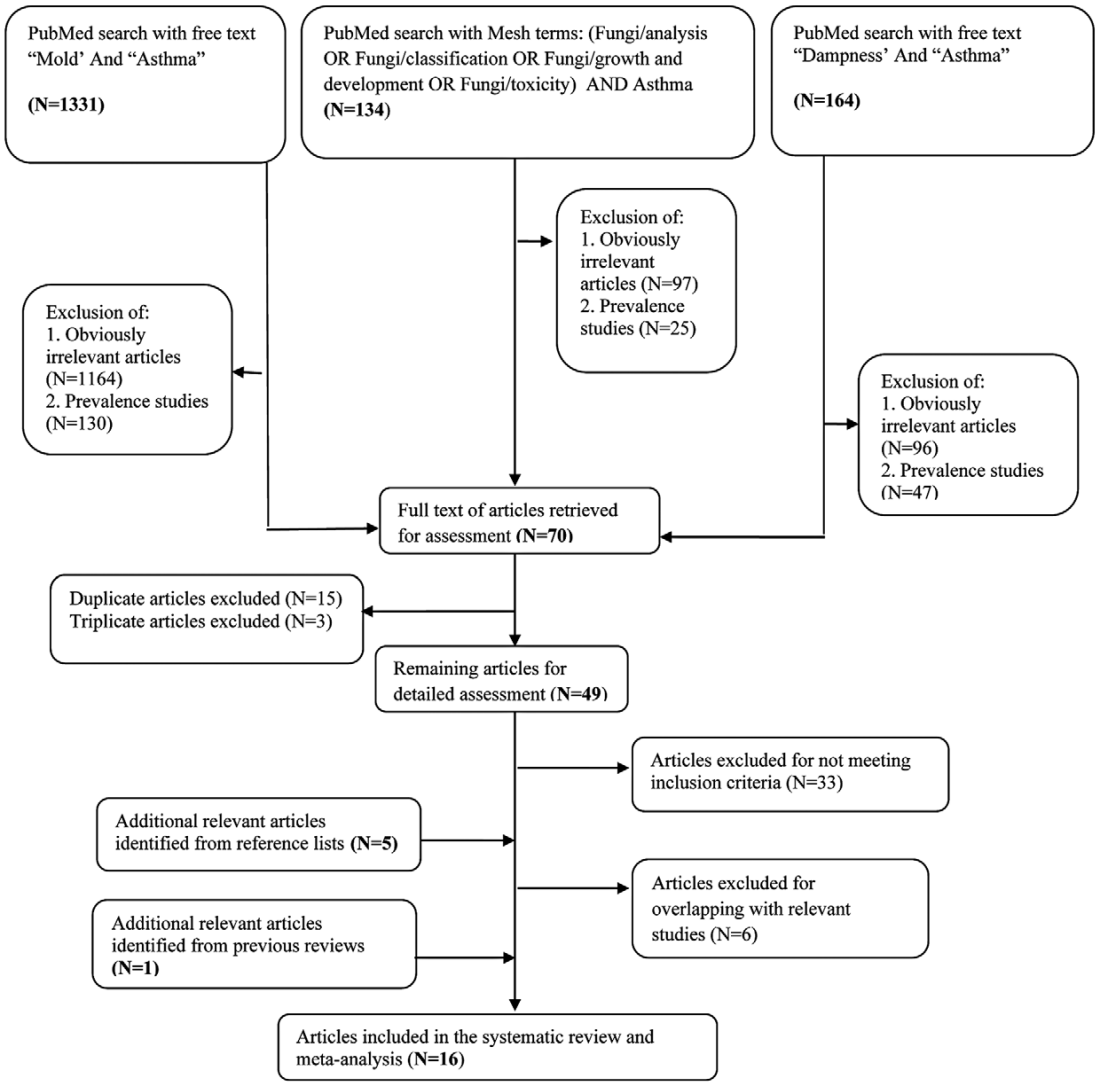
Diagram of the systematic search showing selection of the studies.

We performed a systematic literature search of PubMed database from 1990 through March 2012, as described in **Figure** **1**. We further searched the reference lists of recent reviews and of relevant articles identified in our search.

### Eligibility criteria and outcome and exposure definitions

Three authors independently evaluated the articles. Studies that met the following *a priori* criteria were included: the study (i) was an original study, (ii) was a cohort/longitudinal or an incident case-control study, (iii) reported new cases or onset of asthma, (iv) included a study population of children/infants or adults or both, (v) reported on the relations between dampness and/or mold exposure and new asthma, and (vi) reported dampness and/or mold exposure in the home environment. A study was excluded if the study population included asthmatics at baseline (cohort studies) or prevalent cases (case-control studies). If more than one report was published from the same study, the most recent study or the study with the longest follow-up or the study providing best assessment of exposure and/or outcome was included. The outcome of interest was onset of asthma/development of new asthma. The definitions of asthma considered eligible included the doctor-diagnosis, asthma based on lung function measurements, asthma reported by the patient or parent(s)/guardian(s), reported wheezing, and use of asthma medication. The definitions of exposure that were eligible included water damage, damp stains or other dampness indicators, visible mold, and mold odor.

### Data extraction and quality assessment

The eligible studies were examined and the relevant characteristics of each study recorded in our standard data extraction form [9] independently by the three reviewers. Any disagreements were discussed until a consensus was achieved. **Table** **1** displays the main characteristics of the eligible studies. The study quality was assessed using the Newcastle-Ottawa Scale (NOS) [10] with the maximum score of 9. In the main analysis, studies scoring 8 or 9 were categorized as high quality.

**Table 1:** Characteristics of studies included in the meta-analysis (n = 16).

Reference, Study Year (Country)	Study Population	Study Design	Study Size	Follow-up or Recruitment Period	Definition of Asthma	Method of Data Collection for Exposure Assessment	Total Score on the New Castle-Ottawa Scale (NOS)
Nafstad P [13] 1998 (Norway)	Infants	Incident case-control	Cases: 251, Controls: 251	2 years	Bronchial obstruction diagnosed by a pediatrician, reported by family physician, identified from medical records	Home inspection, parent report confirmed by professional building engineer	7/9
Gent JF [14] 2002 (USA)	Infants	Cohort	880	1 year	Wheezing reported by the mothers in a telephone interview and on a questionnaire	Self-administered questionnaire and airborne mold sampling	9/9
Jaakkola MS [6] 2002 (Finland)	21–63 year old adults	Incident case- control	Cases: 521, Controls: 932	2.5 years	Doctor-diagnosed asthma, including asthma symptoms and lung function measurements	Self-administered questionnaire	8/9
McConnell R [15] 2002 (USA)	9–11 year old children	Cohort	3,535	5 years	Doctor-diagnosed asthma reported in a questionnaire	Self- and interviewer-administered questionnaire	7/9
Rönmark E [16] 2002 (Sweden)	7–8 year old children	Cohort	3,247	2 years	Doctor-diagnosed asthma reported in a questionnaire	Self-administered questionnaire	6/9
Belanger K [17] 2003 (USA)	2–4 month old infants	Cohort	849	1 year	Self-report of wheezing in a telephone interview	Self-administered questionnaire, dust and airborne mold sampling	9/9
Emenius G [18] 2004 (Sweden)	Infants	Incident case- control	Cases: 181, Controls: 359	2 years	Recurrent wheezing reported in a questionnaire	Home inspection by environmental health officers, self-administered questionnaire	7/9
Jaakkola JJK [19] 2005 (Finland)	1-7 year old children	Cohort	1,916	6 years	Doctor-diagnosed asthma reported in a questionnaire	Self-administered questionnaire	9/9
Gunnbjörnsdottir MI [20] 2006 (Iceland, Norway, Sweden, Denmark, Estonia)	Adults	Cohort	15,995	8 years	Asthma attack and/or the use of asthma medication reported in a questionnaire in the second survey	Self-administered questionnaire	8/9
Pekkanen J [21] 2007 (Finland)	Cases 12–84 months old; controls 12–92 months old	Incident case- control	Cases: 121, Controls: 241	Approximately 4 years	Doctor-diagnosed asthma	Home inspection by two trained building engineers	7/9
Karvonen AM [22] 2009 (Finland)	Infants	Cohort	396	1.5 years	Doctor-diagnosed obstructive or asthmatic bronchitis and/or asthma	Home inspection by trained building engineers	8/9
Rosenbaum PF [23] 2010 (USA)	Infants	Cohort	103	2 years	Nurse-diagnosed wheezing	Home inspection by trained field team and airborne mold sampling	9/9
Schroer PF [24] 2009 (USA)	12–14 month old infants	Cohort	570	2 years	Persistent wheezing reported in a questionnaire	Home inspection by trained professional	7/9
Hwang BF [25] 2011 (Taiwan)	Children	Incident case- control	Cases: 188, Controls: 376	6 years	Self-reported doctor diagnosis	Self-administered questionnaire	7/9
Larsson M [26] 2011 (Sweden)	6–8 year old children	Cohort	4,799	5 years	Doctor-diagnosed asthma reported in a questionnaire	Self-administered questionnaire	7/9
Reponen T [27] 2011 (USA)	Children	Cohort	176	7 years	Lung function measurements, caregiver report of asthma symptoms	Biological analysis of mold in dust sample (EMRI) and home inspection	8/9

### Statistical methods

In the meta-analysis we calculated summary effect estimates (EE) from the study-specific odds ratios (OR) and incidence rate ratios (IRR) by using fixed- and random-effects models [11]. When available, we preferred the adjusted effect estimates over the crude estimates. The summary effect estimate from the fixed-effects model is presented when the study-specific effect estimates were homogeneous, whereas that from the random-effects model is presented when moderate or substantial heterogeneity was observed. Heterogeneity was evaluated using the *Q*- and *I^2^*-statistics (*I^2^*-statistic >50% indicates high, 25–50% medium, and <25% low heterogeneity). Stratified and meta-regression analyses were performed to elaborate heterogeneity between study-specific effect estimates. The possibility of publication bias was explored with a funnel plot. We used the “metan” command to run the fixed- and random-effects models on Stata 11 (StataCorp LP, College Station, TX, USA) [12].

## Results

### Literature search

A step-by-step approach of the literature search is shown in **Figure** **1**. Sixteen studies [6], [13]–[27] met the *a priori* inclusion criteria and were included in the systematic review and meta-analysis. Among the 16 articles, 5 were identified from reference lists of relevant studies and 1 from a review by the World Health Organization (WHO) [3]. Thirty-nine articles [28]–[66] were excluded for reasons given in **Table** **S1**. Six of the 16 studies specifically studied the relation between any dampness or mold exposure and onset of asthma, 8 reported on water damage and onset of asthma, 9 on dampness and onset of asthma, 12 on visible mold and onset of asthma, and 8 on mold odor and onset of asthma (**Table** **2**).

**Table 2:** Study-specific and summary effect estimates from the studies included in the meta-analysis using the highest effect estimates reported in the studies.

Reference, Year, Country	Exposure measures and effect estimates (EE)				
	Any exposure EE (95%CI)	Water damage EE (95%CI)	Dampness EE (95%CI)	Visible mold EE (95%CI)	Mold odor EE (95%CI)
Nafstad P [13] 1998 Norway	3.8 (2.0–7.2)^b^				
Gent JF [14] 2002 USA	1.23 (0.94–1.61)^c^	1.18 (0.90–1.55)		1.23 (0.94–1.61)^a^	
Jaakkola MS [6] 2002 Finland	1.02 (0.73–1.41)^c^	0.90 (0.61–1.34)	1.02 (0.73–1.41)	0.98 (0.68–1.40)	0.98 (0.68–1.40)
McConnell R [15] 2002 USA	1.08 (0.78–1.49)^c^	1.08 (0.78–1.49)		0.87 (0.68–1.12)	
Rönmark E [16] 2002 Sweden	1.17 (0.58–2.40)^ c^		1.17 (0.58–2.40)		
Belanger K [17] 2003 USA	1.54 (1.09–2.18)^ c^			1.54 (1.09–2.18)	
Emenius G [18] 2004 Sweden	2.0 (1.20–3.40)^b^		1.50 (1.00–2.30)	1.00 (0.50–1.70)	2.00 (1.00–3.90)
Jaakkola JJK [19] 2005 Finland*	1.01 (0.66–1.54)^b^	1.01 (0.45–2.26)	0.92 (0.54–1.54)	0.65 (0.24–1.72)	2.44 (1.07–5.60)
Gunnbjörnsdottir MI [20] 2006 (Iceland, Norway Sweden, Denmark, Estonia)	1.27 (1.06–1.52)^b^	1.18 (0.95–1.44)	1.67 (1.22–2.27)	1.53 (1.18–1.98)	
Pekkanen J [21] 2007 Finland	4.01 (1.12–14.32)^ c^		1.97 (1.00–3.90)	4.01 (1.12–14.32)	2.96 (0.62–14.19)
Karvonen AM [22] 2009 Finland	5.22 (1.48–18.35)^ c^		1.29 (0.50–3.32)	5.22 (1.48–18.35)	0.66 (0.14–3.17)
Rosenbaum PF [23] 2010 USA	1.32 (0.58–3.22)^c^	1.32 (0.58–3.22)^a^	1.32 (0.58–3.02)^a^	0.90 (0.35–2.29)^a^	1.32 (0.58–3.02)^a^
Schroer KT [24] 2009 USA	2.47 (1.27–4.80)^b^				
Hwang BF [25] 2011 Taiwan	1.69 (1.67–2.45)^b^	2.80 (0.59–13.3)		1.76 (1.18–2.26)	2.09 (1.30–3.37)
Larsson M [26] 2011 Sweden ^µ^	2.99 (1.50–5.94)^c^	0.93 (0.55–1.57)	1.49 (0.35–6.30)	1.49 (0.35–6.30)	2.99 (1.50–5.94)
Reponen T [27] 2011 USA	0.88 (0.52–1.48)^c^				
*Q* statistics, *P* value	38.74, 0.001	3.65, 0.819	8.22, 0.413	30.30, 0.001	14.85, 0.038
*I^2^*-index	61.3	0.0	2.6	63.7	52.9
Summary Effect Estimate					
Fixed-Effects Model	1.35 (1.23–1.49)	1.12 (0.98–1.27)	1.33 (1.12–1.56)	1.27 (1.14–1.41)	1.56 (1.25–1.95)
Random-Effects Model	1.50 (1.25–1.80)	1.12 (0.98–1.27)	1.32 (1.12–1.57)	1.29 (1.04–1.60)	1.73 (1.19–2.50)

EE, effect estimate, either odds ratio or incidence rate ratio; Cl, confidence interval; ^µ^, reported effect estimate for dampness and visible mold; ^*^, reported effect estimate for visible mold and mold odor; ^a^, unadjusted OR; ^b^, estimates for any exposure indicator reported in the studies; ^c^, highest effect estimates reported in the studies and used as any exposure indicator in the meta-analysis.

### Design characteristics of included studies

Characteristics of the 16 eligible studies are shown in **Table** **1**. Definition of asthma was based on lung function measurements, doctor-diagnosed asthma by clinical examination, reported doctor-diagnosis, reporting of asthma attacks and/or the use of asthma medication, and reporting of wheezing and signs of asthma (**Table** **1**).

Information on exposure was reported by home occupants in questionnaires or in an interview or by trained/professional inspectors. The studies defined exposures in variable ways (**Table** **S2**) and we systematically categorized them into any exposure, water damage, dampness, visible mold, and mold odor (**Table** **2**). Six of the 16 studies provided effect estimates for the relation between any exposure and onset of asthma. For 10 studies, the effect estimate for ‘any exposure’ was chosen or calculated based on the effect estimates reported for specific exposure indicators. Analyses were conducted based on both the highest (**Table** **2**) and the lowest effect estimates (**Table** **S3**) for specific exposures.

### Any exposure and onset of asthma

**Figure 2:**
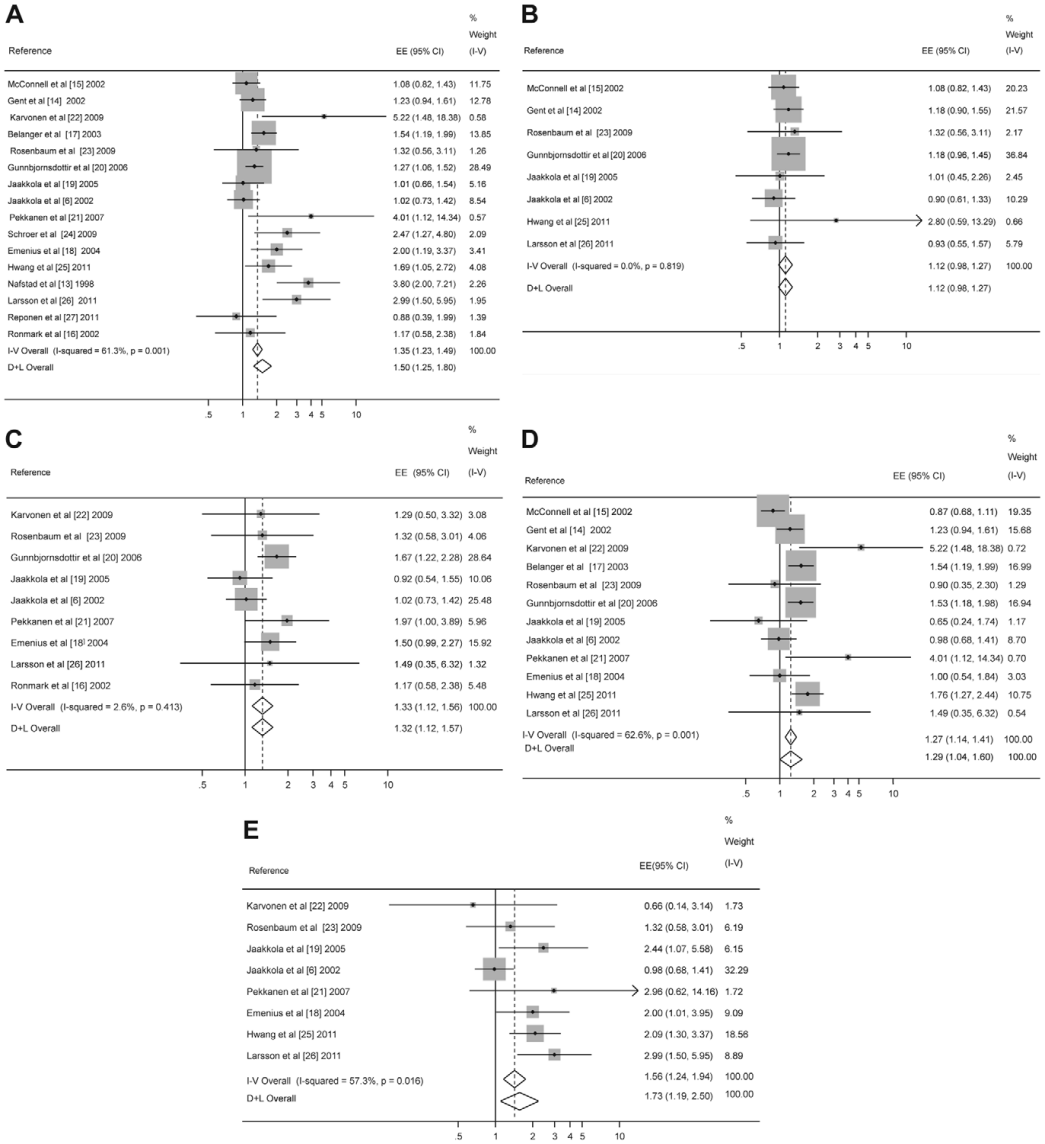
Forest plots. **A.** Forest plot for the relation between any exposure and onset of asthma (n = 16). **B.** Forest plot for the relation between water damage and onset of asthma (n = 8). **C.** Forest plot for the relation between dampness and onset of asthma (n = 9). **D.** Forest plot for the relation between visible mold and onset of asthma (n = 12). **E.** Forest plot for the relation between mold odour and onset of asthma (n = 8).

**Figure** **2A** shows the forest plot of 16 effect estimates for the relation between any dampness or mold exposure and onset of asthma. The summary effect estimate from the random-effects model was 1.50 (95% CI 1.25–1.80) based on the highest study-specific effect estimates (**Table** **S4**) and 1.31 (95% CI 1.09–1.58) based on the lowest effect estimates (**Table** **S5**). Substantial heterogeneity was observed in the study-specific effect estimates. We elaborated sources of heterogeneity by conducting stratified analyses (**Tables** **S4** and **S5**). The summary effect estimate (EE) based on the highest study-specific effect estimates was the highest among infants (1.99), the second highest among children (1.30) and lowest among adults (1.19). The analyses stratified by study design showed only slight differences in the effect estimate based on cohort (1.36, 1.14–1.63) vs. incident case-control studies (1.97, 1.19–3.28). The effect estimate based on studies using home inspection for exposure assessment was substantially higher (2.24, 1.47–3.41) compared with the estimate based on studies with self-reported exposure (1.29, 1.11–1.48; **Table** **S4**). However, both effect estimates were statistically significant. Exposure assessment remained a significant determinant of heterogeneity also in the meta-regression indicating that it has an important independent role in producing heterogeneity between studies. On the other hand, the fact that the higher quality assessment (home inspection) produces stronger effect estimates strengthens the conclusions about the studied relation. Very limited number of studies provided any measurements of fungi or related compounds, so there was not enough data to carry out a meta-analysis based on them. In meta-regression analysis, infant population (*P* = 0.086), exposure assessment method (*P* = 0.020), and study quality (*P* = 0.061) were determinants of heterogeneity, while climatic zone (*P* = 0.546) or other covariates did not explain heterogeneity.

The funnel plot was moderately asymmetric indicating a possibility of publication bias (**Figure** **S1**). Adjustment for publication bias by imputing four “missing” studies using the trim and fill method reduced marginally the strength of the overall summary effect estimate to 1.31 (95% CI 1.07–1.65).

### Water damage and onset of asthma

The summary effect estimate for water damage, based on 8 available homogeneous effect estimates (**Figure** **2B**), was slightly, but not significantly elevated (fixed-effects model; EE 1.12, 95% CI 0.98–1.27).

### Dampness and onset of asthma

The relation between dampness indicators and onset of asthma was reported in 9 studies (**Figure** **2C**). The summary effect estimate, based on rather homogeneous study-specific effect estimates, was significantly increased, being 1.33 (fixed-effects model; 95% CI 1.12–1.56). There was no evidence of publication bias. The stratified analyses are presented in **Table** **S6**.

### Visible mold and onset of asthma

Twelve studies estimated the relation between visible mold and onset of asthma (**Figure** **2D**). The meta-analysis based on the highest study-specific effect estimates gave the summary effect estimate of 1.29 (random-effects model; 95% CI 1.04–1.60). There was no evidence of publication bias. There was substantial heterogeneity between study-specific estimates, but the stratified analysis and meta-regression did not identify any significant determinant of this (**Table** **S7**).

### Mold odor and onset of asthma

The relation between mold odor and onset of asthma was reported in 8 studies (**Figure** **2E**). The random-effects model provided a significantly increased summary effect estimate of 1.73 (95% CI 1.19–2.50). There was no evidence of publication bias. There was substantial between-study variability. In the stratified analyses (**Table** **S8**), the effect estimate was higher in studies with long follow-up (1.91) compared to those of shorter duration (1.20) and in studies conducted in continental cool summer zone (EE 1.59, 95% CI 1.01–2.81).

## Discussion

**Figure 3:**
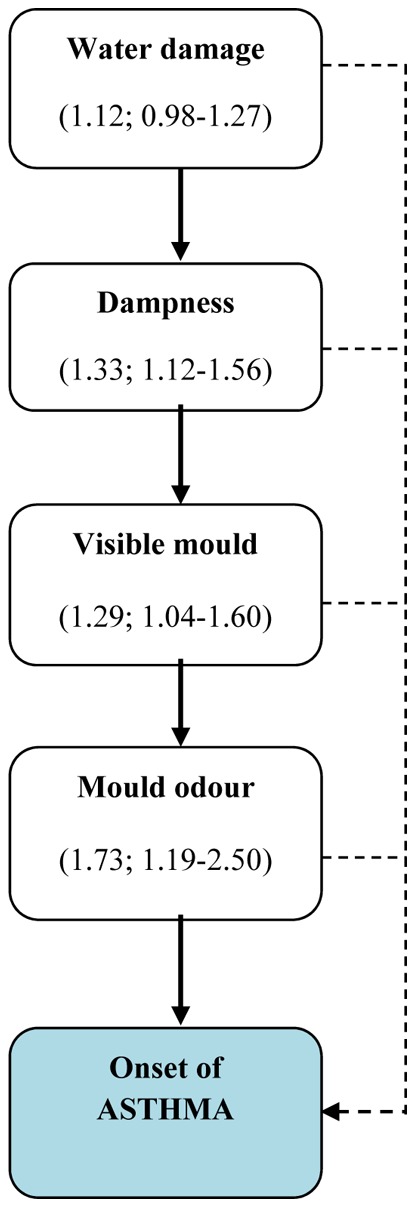
Effect estimates for exposure indicators according to the hypothesized simplified causal pathway.

This systematic review and meta-analysis clearly demonstrates that the risk of developing asthma is significantly increased in relation to home dampness and molds present prior to the onset of disease. In order to strengthen the causal inference and improve the quantification of the effect size, we focused on the strongest study designs including only cohort and incident case-control studies with new cases of asthma, and therefore assessing exposure that took place prior to the onset/diagnosis of disease. Further we elaborated different exposure indicators which characterize exposure at different points of a hypothesized simplified causal pathway starting from the occurrence of water damage or leakages, followed by the presence of dampness on indoor surfaces and structures, and finally, the presence of visible mold and sensation of mold odor in the home (**Figure** **3**).

To quantify the overall risk we identified or constructed an exposure indicator for any exposure which allowed us to calculate a summary effect estimate based on all 16 eligible epidemiologic studies. The summary effect estimate for any exposure to home dampness or molds indicated an average 50% increase in the risk of asthma when the highest study-specific effect estimates were applied. The corresponding increase in the risk of asthma was 31% when the lowest study-specific effect estimates were used. Both estimates were statistically significant. The summary effect estimates related to specific exposures were consistently increased for mold odor (an average 73% increase in the risk of asthma), visible mold (29% increase) and dampness (33% increase), while the asthma risk was not significantly increased in relation to water damage *per se*. Interestingly, the exposure assessment based on inspection produced higher risk estimates than self-reports, suggesting that study subjects tend to underestimate their exposure to indoor dampness and mold problems.

### Validity of results

The strengths of our study include selection of individual studies based on a clearly defined search strategy. In addition to the primary PubMed search, we also used secondary references that were cited by the articles and reviews identified in the primary search. Three reviewers checked independently the eligibility of the studies according to *a priori* set inclusion and exclusion criteria and decided on the most appropriate effect estimate. Any disagreements were settled by discussion.

The present systematic review focused on epidemiological studies with strong design including only cohort and incident case-control studies, and therefore all cross-sectional studies were excluded. This was because we wanted to address the study question whether indoor dampness and mold problems are related to development of new asthma, a question that has remained open in previous reviews.

We evaluated the possibility of publication bias using funnel plots and ‘metarim’ method for simulating any asymmetry appearing in funnel plots. There was an indication of slight publication bias when addressing the effect of any dampness-related exposure. However, addition of four studies to eliminate the asymmetry of the funnel plot using the trim and fill method reduced the strength of the overall effect estimate marginally from 1.50 to 1.31, but the effect remained statistically significant (95% CI 1.07–1.65). Publication bias diagnostics for the four specific exposure indicators showed no suggestion of publication bias.

### Synthesis with previous knowledge

One previous meta-analysis [5] has addressed the relations between any exposure to indoor dampness or molds and asthma, but it focused on prevalent asthma, i.e. ever-diagnosed asthma or current asthma, finding a significant excess risk of 37–56%. Our summary effect estimate for the relation between any dampness or mold and onset of asthma was 1.50 based on the highest reported estimates and 1.31 based on the lowest reported estimates. Interestingly the estimated excess risk was most consistent and highest in relation to mold odor at home, with an excess risk of 73%. This could mean that mold odor is the most specific exposure related to asthma, as it typically refers to microbial contamination of indoor space. Being able to smell odor indicates that there is a connection between the damaged area and the breathing zone of people occupying that space. It also usually reflects long-term exposure to dampness, as it takes some time until there is enough microbial growth to produce enough chemical compounds to be sensed as odor. A study [50] from USA investigated asthma that was diagnosed by a physician after the employees had worked in a large office building with a history of water damage. They measured microbes in floor dust and found significantly increased risk of asthma in relation to exposure to hydrophilic fungi, with an OR 2.19 (95% CI 1.23–3.89), as well as in relation to yeast exposure, with an OR1.77 (95% CI 1.05–3.01), supporting the hypothesis that indoor microbial growth plays a major role in the development of asthma. In our meta-analysis, visible mold was also significantly related to an increased risk of developing asthma, although the effect estimate was somewhat lower (EE 1.29, 95% CI 1.04-1.60) than the estimate for mold odor (EE 1.73, 95% CI 1.19–2.50) and there was more heterogeneity. A population-based incident case-control study from Finland [6] showed a significantly increased risk of adult-onset asthma in relation to visible mold or mold odor at work, with an OR of 1.54 (95% CI 1.01–2.32), which is in line with our findings related to home exposure.

Toxicologic studies have suggested plausible biologic mechanisms demonstrating inflammatory, cytotoxic and immunosuppressive responses to exposure to spores, and metabolites and components of different microbial species. These were recently reviewed by WHO Europe [3] and Mendell et al [8]. It has been suggested that different species of microbes have their effect through different mechanisms, including an IgE-mediated hypersensitivity reaction [34], other immunologic reactions, cytotoxic reactions, and irritant inflammatory effects caused by cell wall components, such as 1,3-ß-D-glucan, endotoxins and extracellular polysaccharides [1], [2], [19], [57], [67]–[69].

Exposure to dampness indicators was related to a smaller, but still significant risk of developing asthma with an effect size of 1.33 (95% CI 1.12–1.56). This may reflect a less extensive damage or a damage of shorter duration, or the role of other exposures than microbes. Several potential causal factors have been suggested to play a role for asthma in relation to dampness problems, including house dust mites and chemicals emitted from damp materials in addition to mold and bacterial growth [2], [19], [70]–[73].

Water damage *per se* was not related to an excess risk of developing asthma (summary effect estimate; 1.12, 95% CI 0.98–1.27) in our meta-analysis, which is logical considering that the first step in the causal pathway starts with the water damage with no specific causal exposures at the beginning (Figure 3). With a prolonged dampness problem potential causal agents emerge, including microbes, house dust mites, and chemical emissions.

Overall our results provide evidence that indoor dampness and mold problems are related to development of the chronic disease condition asthma, which gives weight to the need for preventive and remediation actions. Indeed, our results give ground for the recommendation by a recent expert review suggesting that an intervention of ‘combined elimination of moisture intrusion and leaks and removal of moldy items’ is effective for reducing health risks to occupants [8], [74].

## Conclusions

The evidence up to date from the most valid studies indicates that dampness and mold in the home are determinants of developing asthma. The association of the presence of visible mold and especially mold odor to the risk of asthma points towards mold-related causal agents. Home dampness may also be related to other indoor environmental factors, such as dust mites and chemical emissions from damp structures and surface materials, which may be other causal agents related to development of asthma. Our results provide evidence that justifies prevention and remediation of indoor dampness and mold problems and such actions are likely to reduce induction of new asthma, lead to savings in the health care costs and improvement of public health.

## Supporting Information

Figure S1 **Funnel plot for the relation between any exposure and the onset of asthma (based on the highest effect estimates reported in the studies).**
(DOCX)

Table S1 Studies identified in the search but excluded from the meta-analysis (n = 39).
(DOCX)

Table S2 Definition or description of exposure in the original studies included in the meta-analysis (arranged in the order as classified in Table 2: n = 16).
(DOCX)

Table S3 Effect estimates reported in the studies included in the meta-analysis (the lowest effect estimates reported in the studies).
(DOCX)

Table S4 Summary effect estimates (EEs) for the relation between any exposure (including the highest effect estimates in the studies) and the risk of asthma onset (n = 16) and stratified analysis according to the study characteristics.
(DOCX)

Table S5 Summary effect estimates (EEs) for the relation between any exposure (including the lowest effect estimates in the studies) and the risk of asthma onset (n = 16) and stratified analysis according to the study characteristics.
(DOCX)

Table S6 Summary effect estimates (EEs) for the relation between dampness and the risk of asthma onset (n = 9) and stratified analysis according to the study characteristics.
(DOCX)

Table S7 Summary effect estimates (EEs) for the relation between visible mold and the risk of asthma onset (n = 12) and stratified analysis according to the study characteristics.
(DOCX)

Table S8 Summary effect estimates (EEs) for the relation between mold odor and the risk of asthma onset (n = 8) and stratified analysis according to the study characteristics.
(DOCX)
