# Correction: Residential Dampness and Molds and the Risk of Developing Asthma: A Systematic Review and Meta-analysis

**DOI:** 10.1371/journal.pone.0093454

**Published:** 2014-03-26

**Authors:** The PLOS ONE Staff

There are numerous errors throughout this article.

There are errors in the second sentence of the "Synthesis" subheading of the abstract. The summary effect estimates, the 95% CI and the heterogeneity statistics based on the highest and the lowest estimates for the relation between any exposure and onset of asthma in the original article are incorrect. The correct estimates are: for the highest estimates: 1.48 (95% confidence interval [CI] 1.23-1.78, random-effects model, Q-statistic 38.75 (16), P  =  0.001) ; and for the lowest estimates: 1.27 (95% CI 1.06-1.53, random-effects model, Q-statistic 38.12 (16), P  =  0.000).

Several errors occur in the first paragraph of the Results section under the subheading "Any exposure and onset of asthma":

In the second sentence the summary effect estimate from the random-effects model for the highest estimates and the lowest estimates are incorrect in the original article. The correct summary effect estimate for the highest effect estimates for the random effect model is 1.48 (95% CI 1.23-1.78). The correct summary effect estimate for the lowest effect estimates for the random effect model is 1.27 (95% CI 1.06-1.53).

In the fifth sentence the summary effect estimate from the random-effects model for the highest estimates among adult is incorrect in the original article. The correct summary effect estimate is 1.10.

In the sixth sentence the analysis stratified by study design is incorrect for cohort studies in the original article. The correct summary effect estimate is 1.34 (1.11-1.63).

In the seventh sentence the analysis stratified by exposure assessment is incorrect for self-reported exposure in the original article. The correct summary effect estimate is 1.26 (1.08-1.47).

In the twelfth sentence the p-value for infant in the meta-regression analysis is incorrect in the original article. The correct p-value is 0.058.

Figure 2A is incorrect. The authors have provided a correct version of Figure 2A here.

**Figure 2:**
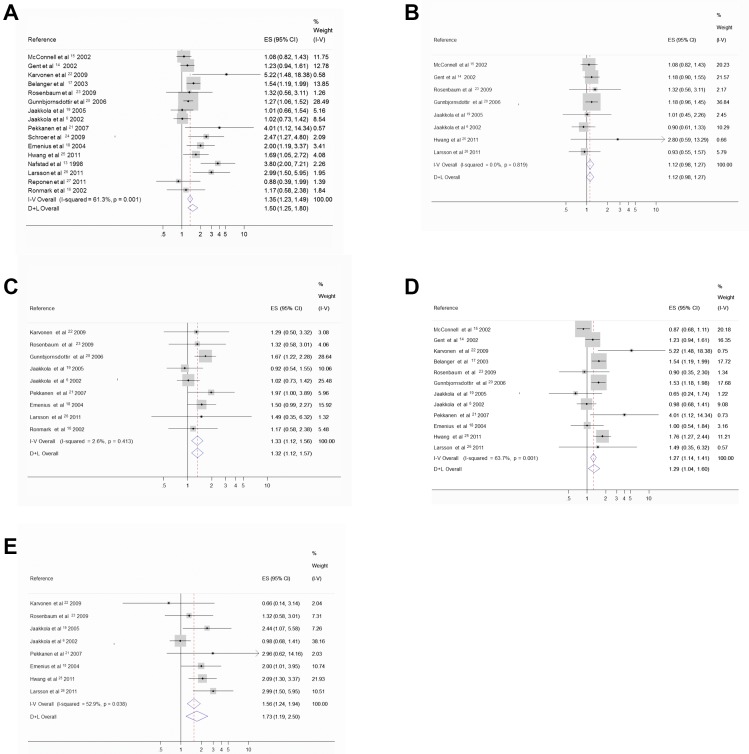
Forest plots. **A.** Forest plot for the relation between any exposure and onset of asthma (n  =  16). **B.** Forest plot for the relation between water damage and onset of asthma (n  =  8). **C.** Forest plot for the relation between dampness and onset of asthma (n  =  9). **D.** Forest plot for the relation between visible mold and onset of asthma (n  =  12). **E.** Forest plot for the relation between mold odour and onset of asthma (n  =  8).

Several errors occur in the Discussion section:

In the second sentence of the second paragraph, the average increase for any exposure to home dampness or molds is incorrect in the original article for the highest study-specific estimates. The correct average increase is 48%.

In the third sentence of the second paragraph, the average increase for any exposure to home dampness or molds is incorrect in the original article for the lowest effect estimates. The correct average increase is 27%.

In the first paragraph of the subheading, "Synthesis with previous knowledge", in the second sentence the summary effect estimates for the relation between dampness or mold and onset of asthma for the highest effect estimates and the summary effect estimates for the relation between dampness or mold and onset of asthma for the lowest effect estimates are incorrect in the original article. The correct estimates are, respectively, 1.48 and 1.27.

SI Tables S3, S4, and S5 are incorrect. The authors have provided correct versions of SI Tables S3, S4, and S5 here.

## Supporting Information

Table S3 Effect estimates reported in the studies included in the meta-analysis (the lowest effect estimates reported in the studies).

Table S4 Summary effect estimates (EEs) for the relation between any exposure (including the highest effect estimates in the studies) and the risk of asthma onset (n  =  16) and stratified analysis according to the study characteristics.

Table S5 Summary effect estimates (EEs) for the relation between any exposure (including the lowest effect estimates in the studies) and the risk of asthma onset (n  =  16) and stratified analysis according to the study characteristics.
