# Supplementary material for: Residential Dampness and Molds and the Risk of Developing Asthma: A Systematic Review and Meta-Analysis
Source: PLoS One. 2012 Nov 7;7(11):e47526. doi: 10.1371/journal.pone.0047526 (PMC3492391; doi:10.1371/journal.pone.0047526)
Supplement: Figure S1 — Funnel plot for the relation between any exposure and the onset of asthma (based on the highest effect estimates reported in the studies). (DOCX) [file pone.0047526.s001.docx]

**Figure S1. Funnel plot for the relation between any exposure and the onset of asthma (based on the highest effect estimates reported in the studies).**
