# Supplementary material for: Residential Dampness and Molds and the Risk of Developing Asthma: A Systematic Review and Meta-Analysis
Source: PLoS One. 2012 Nov 7;7(11):e47526. doi: 10.1371/journal.pone.0047526 (PMC3492391; doi:10.1371/journal.pone.0047526)
Supplement: Table S1 — Studies identified in the search but excluded from the meta-analysis (n = 39). (DOCX) [file pone.0047526.s002.docx]

**Table S1.** Studies identified in the search but excluded from the meta-analysis (n= 39)

| **Reasons for exclusion** | **Excluded studies** |
| --- | --- |
| Cross-sectional studies and prevalent case-control studies | Lee YL [28] 2006; Salo PM [29] 2006; Dales RE [30] 1991 |
| Exposure definition incompatible with the *a priori* criteria of this meta-analysis | Torén K [31] 2011; Matheson MC [32] 2005; Douwes et J [33] 2006; Jaakkola MS [34] 2006; Raphoz M [35] 2010; Klinnert MD [36] 2001; Kozyrskyj AL [37] 2011; Leander M [38] 2009; Immonen J [39] 2001; Jaakkola MS [40] 2002; Halonen M [41] 1997; Korhonen K [42] 2006; Karvala K [43] 2010; Karvala K [44] 2011 |
| Outcome definition incompatible with the *a priori* criteria of this meta-analysis | Dales R [45] 2010; Stark PC [46] 2003; Stark PC [47] 2005; Sahlberg B [48] 2009; Norbäck D [49] 2011; Park JH [50] 2008; Hägerhed-Engman L [51] 2009; Lindfors A [52] 1995; Bundy KW [53] 2009; Pongracic JA [54] 2010; Nguyen T [55] 2010 |
| Unclear from the studies whether new cases of asthma were investigated | Jedrychowski W [56] 2007; Tischer C [57] 2011; Mommers M [58] 2005; Thorn J [59] 2001; Chen YC [60] 2011 |
| Overlap with studies included in this meta-analysis | Iossifova YY [61] 2009; Iossifova YY [62] 2007; Vesper SJ [63] 2007; Cho SH [64] 2006; Wickman M [65] 1992; Hyvärinen A [66] 2006 |
