# Supplementary material for: Residential Dampness and Molds and the Risk of Developing Asthma: A Systematic Review and Meta-Analysis
Source: PLoS One. 2012 Nov 7;7(11):e47526. doi: 10.1371/journal.pone.0047526 (PMC3492391; doi:10.1371/journal.pone.0047526)
Supplement: Table S2 — Definition or description of exposure in the original studies included in the meta-analysis (arranged in the order as classified in Table 2: n = 16). (DOCX) [file pone.0047526.s003.docx]

**Table S2.** Definition or description of exposure in the original studies included in the meta-analysis (arranged in the order as classified in Table 2: n=16)

| **References, study name/country** | **Exposures as defined or described in the studies** |
| --- | --- |
| Nafstad P [13] 1998 Norway | Home dampness problems defined as the presence of water damage, damp stains, or visible mold/mildew growth |
| Gent JF [14] 2002 USA | Water leakage; mold or mildew |
| Jaakkola MS [6] 2002 Finland | Water damage; damp stains or paint peeling; visible mold and/or mold odor |
| McConnell R [15] 2002 USA | Water damage; mold or mildew |
| Belanger K [17] 2003 USA | Persistent mold or mildew in the home living area |
| Rönmark E [16] 2002 Sweden | Dampness |
| Emenius G [18] 2004, Sweden | Any sign of dampness combined with reported window-pane condensation (WPC) > 2 times, absolute indoor humidity above median level 5.8g/kg ^a^; prospectively dampness reported or detected at an inspection; mold spots on surface material/tile joints in wet areas (shower/bath room); mold odor |
| Jaakkola JJK [19] 2005 Finland | Presence of any of the exposure indicators: water damage; wet spots in the ceilings, floors or walls in the dwelling; visible mold; mold odor |
| Gunnbjörnsdottir MI [20] 2006, (Iceland, Norway, Sweden, Denmark, Estonia) | Any dampness; water damage; wet floors; visible mold |
| Pekkanen [21] 2007 Finland | Water damage in child’s bedroom; mold spots in main living area; mold odor in main living area |
| Karvonen AM [22] 2009 Finland | Moisture damage in the child’s bedroom; visible mold in the child’s bedroom; mold odor in the whole house (including child’s bedroom, main living area, kitchen, bathroom) |
| Rosenbaum PF [23] 2010 USA | Visible dampness/water; moldy/musty/damp odor^b^; visible mold |
| Schroer KT [24] 2009 USA | Mold ^c^ |
| Hwang BF [25] 2011, Taiwan | Presence of any of the exposure indicators: water damage; wet spots in the ceilings, floors or walls in the dwelling; visible mold; mold odor |
| Larsson M [26] 2011 Sweden | Flooding or other types of water damage in the child’s birth residence; visible mold or damp stains on the floor^d^, wall or roof in the child’s birth residence; mold odor in the child’s birth residence or perceived earthy odor in child’s birth residence |
| Reponen T [27] 2011 USA | Low mold (reported or observed water damage, observed moldy odor, or visible mold area ≤0.2 m^2^ ); high mold (visible mold area >0.2 m^2^-i.e. at least 0.2 m^2^ of visible mold or mold and water damage combined) |

**Legend**

^a^ Any sign of dampness defined as damp stain/mold-mildew-spots/mold odor, etc; not including mold spots on surfaces in wet areas and window pane condensation; ^b^ exposure to dampness and mold odor; ^c^ mold exposure defined as visible mold, water damage, or moldy odor; ^d^ exposure to dampness and visible mold
