# Supplementary material for: Residential Dampness and Molds and the Risk of Developing Asthma: A Systematic Review and Meta-Analysis
Source: PLoS One. 2012 Nov 7;7(11):e47526. doi: 10.1371/journal.pone.0047526 (PMC3492391; doi:10.1371/journal.pone.0047526)
Supplement: Table S3 — Effect estimates reported in the studies included in the meta-analysis (the lowest effect estimates reported in the studies). (DOCX) [file pone.0047526.s004.docx]

**Table S3.** Effect estimates reported in the studies included in the meta-analysis (the lowest effect estimates reported in the studies)

| **Reference, Year, Country** | **EE for exposure measures** | | | | |
| --- | --- | --- | --- | --- | --- |
|  | **Any exposure**  EE (95%CI) | **Water damage**  EE (95%CI) | **Dampness**  EE (95%CI) | **Visible mold**  EE (95%CI) | **Mold odor**  EE (95%CI) |
| ^~~¥~~^Nafstad P [13] 1998 Norway | 3.8 (2.0-7.2)^b^ |  |  |  |  |
| Gent JF [14] 2002 USA | 1.18 (0.90-1.55)^c^ | 1.18 (0.90-1.55) |  | 1.23 (0.94-1.61) ^a^ |  |
| ^~~¥~~^Jaakkola MS [6] 2002 Finland | 0.90 (0.61-1.34)^c^ | 0.90 (0.61-1.34) | 1.02 (0.73-1.41) | 0.98 (0.68-1.40) | 0.98 (0.68-1.40) |
| McConnell R [15] 2002 USA | 0.87 (0.68-1.12)^c^ | 1.08 (0.78-1.49) |  | 0.87 (0.68-1.12) |  |
| ^~~¥~~^Rönmark E [16] 2002 Sweden | 1.17 (0.58-2.40)^c^ |  | 1.17 (0.58-2.40) |  |  |
| ^~~¥~~^Belanger K [17] 2003 USA | 1.54 (1.09-2.18) ^c^ |  |  | 1.54 (1.09-2.18) |  |
| ^~~¥~~^Emenius G [18] 2004 Sweden | 2.0 (1.20-3.40) ^b^ |  | 1.50 (1.00-2.30) | 1.00 (0.50-1.70) | 2.00 (1.00-3.90) |
| ^~~¤~~^Jaakkola JJK [19] 2005 Finland^*^ | 1.01 (0.66-1.54) ^b^ | 1.01 (0.45-2.26) | 0.92 (0.54-1.54) | 0.65 (0.24-1.72) | 2.44 (1.07-5.60) |
| ^~~¥~~^Gunnbjornsdottir MI [20] 2006 (Iceland, Norway, Sweden, Denmark, Estonia) | 1.27 (1.06-1.52) ^b^ | 1.18 (0.95-1.44) | 1.67 (1.22-2.27) | 1.53 (1.18-1.98) |  |
| ^~~¥~~^Pekkanen J [21] 2007 Finland | 1.97 (1.00-3.90)^c^ |  | 1.97 (1.00-3.90) | 4.01 (1.12-14.32) | 2.96 (0.62-14.19) |
| ^~~¥~~^Karvonen AM [22] 2009 Finland | 0.66 (0.14-3.17)^c^ |  | 1.29 (0.50-3.32) | 5.22 (1.48-18.35) | 0.66 (0.14-3.17) |
| ^~~¥~~^Rosenbaum PF [23] 2010 USA ^β^ | 0.90 (0.35-2.29)^c^ | 1.32 (0.58-3.22) ^a^ | 1.32 (0.58-3.02) ^a^ | 0.90 (0.35-2.29) ^a^ | 1.32 (0.58-3.02) ^a^ |
| ^~~¥~~^Schroer KT [24] 2009 USA | 2.47 (1.27-4.80) ^b^ |  |  |  |  |
| ^~~¥~~^Hwang BF [25] 2011 Taiwan | 1.69 (1.30-3.37) ^b^ | 2.80 (0.59-13.3) |  | 1.76 (1.18-2.26) | 2.09 (1.30-3.37) |
| ^~~¥~~^Larsson M [26] 2011 Sweden µ | 0.93 (0.55-1.57) ^c^ | 0.93 (0.55-1.57) | 1.49 (0.35-6.30) | 1.49 (0.35-6.30) | 2.99 (1.50-5.94) |
| ^~~¥~~^Reponen [27] 2011  USA | 0.88 (0.52-1.48)^c^ |  |  |  |  |

**Legend**

EE, effect estimate, either odds ratio or incidence rate ratio; Cl, confidence interval; ^µ^, reported effect estimate for dampness and visible mold; ^*^, reported effect estimate for visible mold and mold odor; ^β^ , reported effect estimate for dampness and mold odor; ^a^, unadjusted OR; ^b^, estimates for any exposure indicator reported in the studies; ^c^, lowest effect estimates reported in the studies and used as any exposure indicator in the meta-analysis
