# Supplementary material for: Residential Dampness and Molds and the Risk of Developing Asthma: A Systematic Review and Meta-Analysis
Source: PLoS One. 2012 Nov 7;7(11):e47526. doi: 10.1371/journal.pone.0047526 (PMC3492391; doi:10.1371/journal.pone.0047526)
Supplement: Table S4 — Summary effect estimates (EEs) for the relation between any exposure (including the highest effect estimates in the studies) and the risk of asthma onset (n = 16) and stratified analysis according to the study characteristics. (DOCX) [file pone.0047526.s005.docx]

**Table S4.** Summary effect estimates (EEs) for the relation between any exposure (including the highest effect estimates in the studies) and the risk of asthma onset (n=16) and stratified analysis according to the study characteristics

| **Stratification** | **Model** | | | | **Heterogeneity Statistics** | | |
| --- | --- | --- | --- | --- | --- | --- | --- |
|  | **Fixed-effects model**  **EE (95%CI)** | | **Random-effects model**  **EE (95%CI)** | | **Q (n)** | **I^2^- statistics**  **(%)** | **P value** |
| **Main analysis** | 1.35 | 1.23-1.49 | 1.50 | 1.25-1.80 | 38.74 (16) | 61.3 | 0.001 |
| **Stratified analysis** |  |  |  |  |  |  |  |
| ***Study population*** |  |  |  |  |  |  |  |
| Infants (0 to 4 years) | 1.63 | 1.39-1.91 | 1.99 | 1.45-2.73 | 18.63 (8) | 62.4 | 0.009 |
| Children (up to 16 years) | 1.23 | 1.02-1.48 | 1.30 | 1.01-2.02 | 10.43 (6) | 52.1 | 0.064 |
| Adults | 1.21 | 1.03-1.41 | 1.19 | 0.98-1.45 | 1.31 (2) | 23.7 | 0.252 |
| ***Study design*** |  |  |  |  |  |  |  |
| Cohort | 1.31 | 1.18-1.46 | 1.36 | 1.14-1.63 | 19.76 (11) | 49.4 | 0.032 |
| Incident case-control | 1.57 | 1.26-1.96 | 1.97 | 1.19-3.28 | 16.92 (5) | 76.4 | 0.008 |
| ***Study size*^a^** |  |  |  |  |  |  |  |
| Large | 1.40 | 1.24-1.59 | 1.68 | 1.28-2.21 | 29.04 (10) | 69.0 | 0.001 |
| Small | 1.29 | 1.11-1.50 | 1.30 | 1.03-1.65 | 9.05 (6) | 44.7 | 0.107 |
| ***Geographical location*** |  |  |  |  |  |  |  |
| USA | 1.31 | 1.13-1.52 | 1.32 | 1.07-1.62 | 7.94 (6) | 37.1 | 0.159 |
| Europe | 1.37 | 1.20-1.56 | 1.74 | 1.25-2.40 | 29.73 (8) | 73.1 | 0.000 |
| ***Climatic zone*** |  |  |  |  |  |  |  |
| Subarctic | 1.15 | 0.91-1.45 | 1.40 | 0.89-2.20 | 10.11 (5) | 60.4 | 0.039 |
| Continental cool summer | 1.60 | 1.37-1.86 | 1.79 | 1.34-2.39 | 18.53 (8) | 62.2 | 0.010 |
| Other | 1.25 | 1.08-1.44 | 1.25 | 1.05-1.50 | 2.61 (3) | 23.4 | 0.271 |
| ***Follow-up in years*** |  |  |  |  |  |  |  |
| >3 years | 1.27 | 1.11-1.45 | 1.35 | 1.05-1.73 | 13.64 (7) | 56.0 | 0.034 |
| ≤3 years | 1.46 | 1.27-1.68 | 1.66 | 1.26-1.80 | 23.02 (9) | 65.2 | 0.003 |
| ***Exposure assessment method*** |  |  |  |  |  |  |  |
| Home inspection | 2.22 | 1.67-2.94 | 2.24 | 1.47-3.41 | 11.92 (7) | 49.6 | 0.089 |
| Self-report | 1.27 | 1.15-1.41 | 1.29 | 1.11-1.48 | 13.68 (9) | 41.5 | 0.090 |
| ***Definition of asthma*** |  |  |  |  |  |  |  |
| Doctor-diagnosed/lung function measurements | 1.31 | 1.12-1.53 | 1.57 | 1.15-2.13 | 31.34 (11) | 68.1 | 0.001 |
| Self-report | 1.38 | 1.22-1.57 | 1.45 | 1.20-1.74 | 7.11 (5) | 43.7 | 0.130 |
| ***Quality*** |  |  |  |  |  |  |  |
| High (scores 8-9) | 1.26 | 1.13-1.41 | 1.25 | 1.07-1.47 | 10.64 (8) | 34.2 | 0.155 |
| Low (scores < 8) | 1.62 | 1.35-1.95 | 1.99 | 1.38-2.88 | 22.83 (8) | 69.3 | 0.002 |

**Legend**

**^a^**Large study: Cohort studies, n > 700; case-control studies, n > 181, where n= study size.
