# Supplementary material for: Residential Dampness and Molds and the Risk of Developing Asthma: A Systematic Review and Meta-Analysis
Source: PLoS One. 2012 Nov 7;7(11):e47526. doi: 10.1371/journal.pone.0047526 (PMC3492391; doi:10.1371/journal.pone.0047526)
Supplement: Table S5 — Summary effect estimates (EEs) for the relation between any exposure (including the lowest effect estimates in the studies) and the risk of asthma onset (n = 16) and stratified analysis according to the study characteristics. (DOCX) [file pone.0047526.s006.docx]

**Table S5.** Summary effect estimates (EEs) for the relation between any exposure (including the lowest effect estimates in the studies) and the risk of asthma onset (n=16) and stratified analysis according to the study characteristics

| **Stratification** | **Model** | | | | **Heterogeneity Statistics** | | |
| --- | --- | --- | --- | --- | --- | --- | --- |
|  | **Fixed-effects model**  **EE (95%CI)** | | **Random-effects model**  **EE (95%CI)** | | **Q (n)** | **I^2^- statistics**  **(%)** | **P value** |
| **Main analysis** | 1.24 | 1.13-1.37 | 1.31 | 1.09-1.58 | 40.08 (16) | 65.1 | 0.000 |
| **Stratified analysis** |  |  |  |  |  |  |  |
| ***Study population*** |  |  |  |  |  |  |  |
| Infants (0-4 years) | 1.54 | 1.31-1.81 | 1.69 | 1.26-2.27 | 17.38 (8) | 59.7 | 0.015 |
| Children (up to 16 years) | 1.00 | 0.84-1.18 | 1.03 | 0.83-1.26 | 6.29 (6) | 20.5 | 0.274 |
| Adults | 1.20 | 1.02-1.41 | 1.12 | 0.81-1.55 | 2.43 (2) | 58.9 | 0.119 |
| ***Study design*** |  |  |  |  |  |  |  |
| Cohort | 1.18 | 1.06-1.31 | 1.16 | 0.99-1.37 | 16.33 (5) | 75.5 | 0.003 |
| Incident case-control | 1.59 | 1.27-2.00 | 1.80 | 1.12-2.90 | 18.27 (11) | 45.3 | 0.051 |
| ***Study size*^a^** |  |  |  |  |  |  |  |
| Large | 1.34 | 1.18-1.52 | 1.47 | 1.14-1.88 | 25.83 (10) | 65.2 | 0.002 |
| Small | 1.13 | 0.98-1.30 | 1.10 | 0.85-1.42 | 11.17 (6) | 55.2 | 0.048 |
| ***Geographical location*** |  |  |  |  |  |  |  |
| USA | 1.17 | 1.02-1.35 | 1.21 | 0.91-1.62 | 15.77 (6) | 68.3 | 0.008 |
| Europe | 1.28 | 1.12-1.46 | 1.34 | 1.02-1.76 | 21.92 (9) | 63.5 | 0.005 |
| ***Climatic zone*** |  |  |  |  |  |  |  |
| Subarctic | 1.12 | 0.71-1.43 | 1.30 | 0.79-2.01 | 10.11 (5) | 60.4 | 0.059 |
| Continental cool summer | 1.60 | 1.37-1.86 | 1.79 | 1.34-2.39 | 18.53 (8) | 62.2 | 0.010 |
| Other | 1.25 | 1.08-1.44 | 1.25 | 1.05-1.50 | 2.61 (3) | 23.4 | 0.271 |
| ***Follow-up in years*** |  |  |  |  |  |  |  |
| >3 years | 1.14 | 1.01-1.29 | 1.14 | 0.92-1.42 | 12.55 (7) | 52.5 | 0.051 |
| ≤3 years | 1.40 | 1.21-1.62 | 1.47 | 1.11-1.96 | 22.99 (9) | 65.2 | 0.003 |
| ***Exposure assessment method*** |  |  |  |  |  |  |  |
| Home inspection | 1.89 | 1.45-2.48 | 1.75 | 1.15-2.65 | 13.25 (7) | 57.1 | 0.034 |
| Self-report | 1.17 | 1.06-1.29 | 1.15 | 0.99-1.35 | 16.09 (9) | 50.3 | 0.041 |
| ***Definition of asthma*** |  |  |  |  |  |  |  |
| Doctor-diagnosed/lung function measurements | 1.08 | 0.93-1.25 | 1.19 | 0.91-1.56 | 26.36 (11) | 62.1 | 0.003 |
| Self-report | 1.37 | 1.21-1.55 | 1.44 | 1.19-1.75 | 7.67 (5) | 47.9 | 0.104 |
| ***Quality*** |  |  |  |  |  |  |  |
| High (scores 8-9) | 1.22 | 1.09-1.37 | 1.20 | 1.05-1.38 | 8.12 (8) | 13.8 | 0.322 |
| Low (scores < 8) | 1.28 | 1.09-1.51 | 1.61 | 1.10-2.37 | 31.77 (8) | 78.0 | 0.000 |

**Legend**

**^a^**Large study: Cohort studies, n > 700; case-control studies, n > 181, where n= study size.
