# Supplementary material for: Residential Dampness and Molds and the Risk of Developing Asthma: A Systematic Review and Meta-Analysis
Source: PLoS One. 2012 Nov 7;7(11):e47526. doi: 10.1371/journal.pone.0047526 (PMC3492391; doi:10.1371/journal.pone.0047526)
Supplement: Table S6 — Summary effect estimates (EEs) for the relation between dampness and the risk of asthma onset (n = 9) and stratified analysis according to the study characteristics. (DOCX) [file pone.0047526.s007.docx]

**Table S6.** Summary effect estimates (EEs) for the relation between dampness and the risk of asthma onset (n=9) and stratified analysis according to the study characteristics

| **Stratification** | **Model** | | | | **Heterogeneity Statistics** | | |
| --- | --- | --- | --- | --- | --- | --- | --- |
|  | **Fixed-effects model**  **EE (95%CI)** | | **Random-effects model**  **EE (95%CI)** | | **Q (n)** | **I^2^- statistics**  **(%)** | **P value** |
| **Main analysis** | 1.33 | 1.12-1.56 | 1.32 | 1.12-1.56 | 8.22 (9) | 2.6 | 0.413 |
| **Stratified analysis** |  |  |  |  |  |  |  |
| ***Study population*** |  |  |  |  |  |  |  |
| Infants (0 to 4 years) | 1.53 | 1.13-2.09 | 1.53 | 1.13-2.09 | 0.79 (4) | 0.0 | 0.853 |
| Children (up to 16 years) | 1.03 | 0.68-1.55 | 1.03 | 0.68-1.55 | 0.55 (3) | 0.0 | 0.759 |
| Adults | 1.32 | 1.06-1.66 | 1.31 | 0.81-2.12 | 4.56 (2) | 78.1 | 0.033 |
| ***Study design*** |  |  |  |  |  |  |  |
| Cohort | 1.39 | 1.10-1.74 | 1.39 | 1.10-1.74 | 4.00 (6) | 0.0 | 0.550 |
| Incident case-control | 1.26 | 0.99-1.61 | 1.34 | 0.93-1.92 | 3.91 (3) | 48.8 | 0.141 |
| ***Study size*^a^** |  |  |  |  |  |  |  |
| Large | 1.33 | 1.12-1.58 | 1.32 | 1.07-1.64 | 8.21 (7) | 26.9 | 0.223 |
| Small | 1.31 | 0.70-2.43 | 1.31 | 0.70-2.43 | 0.0 (2) | 0.0 | 0.971 |
| ***Geographical location*** |  |  |  |  |  |  |  |
| USA | - |  |  |  |  |  |  |
| Europe | 1.33 | 1.12-1.57 | 1.32 | 1.09-1.60 | 8.22 (8) | 14.8 | 0.314 |
| ***Climatic zone*** |  |  |  |  |  |  |  |
| Subarctic | 1.11 | 0.88-1.41 | 1.11 | 0.88-1.41 | 3.59 (5) | 0.0 | 0.464 |
| Continental cool summer | 1.46 | 1.62-2.10 | 1.46 | 1.62-2.10 | 0.07 (3) | 0.0 | 0.964 |
| ***Follow-up in years*** |  |  |  |  |  |  |  |
| >3 years | 1.49 | 1.17-1.91 | 1.45 | 1.03-2.05 | 4.42 (4) | 32.1 | 0.220 |
| ≤3 years | 1.20 | 0.96-1.50 | 1.20 | 0.96-1.50 | 2.12 (5) | 0.0 | 0.714 |
| ***Exposure assessment method*** |  |  |  |  |  |  |  |
| Home inspection | 1.53 | 1.13-2.09 | 1.53 | 1.13-2.09 | 0.79 (4) | 0.0 | 0.853 |
| Self-report | 1.25 | 1.03-1.52 | 1.22 | 0.93-1.60 | 6.22 (5) | 35.6 | 0.184 |
| ***Definition of asthma*** |  |  |  |  |  |  |  |
| Doctor-diagnosed/lung function measurements | 1.14 | 0.91-1.42 | 1.14 | 0.91-1.42 | 3.89 (7) | 0.0 | 0.692 |
| Self-report | 1.61 | 1.25-2.06 | 1.61 | 1.25-2.06 | 0.16 (2) | 0.0 | 0.685 |
| ***Quality*** |  |  |  |  |  |  |  |
| High (scores 8-9) | 1.26 | 1.03-1.53 | 1.23 | 0.94-1.61 | 6.83 (6) | 34.9 | 0.189 |
| Low (scores < 8) | 1.51 | 1.11-2.06 | 1.51 | 1.11-2.06 | 1.08 (4) | 0.0 | 0.233 |

**Legend**

^a^ Large study: Cohort studies n > 700; case-control studies n > 181, where n = study size
