# Supplementary material for: Residential Dampness and Molds and the Risk of Developing Asthma: A Systematic Review and Meta-Analysis
Source: PLoS One. 2012 Nov 7;7(11):e47526. doi: 10.1371/journal.pone.0047526 (PMC3492391; doi:10.1371/journal.pone.0047526)
Supplement: Table S7 — Summary effect estimates (EEs) for the relation between visible mold and the risk of asthma onset (n = 12) and stratified analysis according to the study characteristics. (DOCX) [file pone.0047526.s008.docx]

**Table S7.** Summary effect estimates (EEs) for the relation between visible mold and the risk of asthma onset (n=12) and stratified analysis according to the study characteristics

| **Stratification** | **Model** | | | | **Heterogeneity Statistics** | | |
| --- | --- | --- | --- | --- | --- | --- | --- |
|  | **Fixed-effects model**  **EE (95%CI)** | | **Random-effects model**  **EE (95%CI)** | | **Q (n)** | **I^2^- statistics**  **(%)** | **P value** |
| **Main analysis** | 1.27 | 1.14-1.41 | 1.29 | 1.04-1.60 | 30.30 (12) | 63.7 | 0.001 |
| **Stratified analysis** |  |  |  |  |  |  |  |
| ***Study population*** |  |  |  |  |  |  |  |
| Infants (0 to 4 years) | 1.39 | 1.17-1.65 | 1.44 | 1.05-1.97 | 10.23 (6) | 51.1 | 0.069 |
| Children (up to 16 years) | 1.07 | 0.90-1.28 | 1.07 | 0.71-1.62 | 13.54 (4) | 70.5 | 0.009 |
| Adults | 1.32 | 1.07-1.62 | 1.25 | 0.81-1.93 | 3.86 (2) | 74.1 | 0.049 |
| ***Study design*** |  |  |  |  |  |  |  |
| Cohort | 1.22 | 1.08-1.38 | 1.21 | 0.95-1.55 | 22.22 (8) | 64.0 | 0.005 |
| Incident case-control | 1.35 | 1.08-1.68 | 1.38 | 0.88-2.16 | 9.31 (4) | 67.8 | 0.025 |
| ***Study size* ^a^** |  |  |  |  |  |  |  |
| Large | 1.39 | 1.18-1.64 | 1.33 | 1.00-1.77 | 12.22 (6) | 50.9 | 0.057 |
| Small | 1.16 | 1.01-1.33 | 1.19 | 0.87-1.61 | 17.05 (6) | 70.7 | 0.004 |
| ***Geographical location*** |  |  |  |  |  |  |  |
| USA | 1.32 | 1.10-1.60 | 1.36 | 0.93-1.99 | 11.4 7 (4) | 65.1 | 0.022 |
| Europe | 1.13 | 0.99-1.31 | 1.11 | 0.85-1.45 | 14.17 (7) | 57.7 | 0.028 |
| ***Climatic zone*** |  |  |  |  |  |  |  |
| Subarctic | 1.14 | 0.83-1.57 | 1.67 | 0.69-4.03 | 11.28 (4) | 73.4 | 0.010 |
| Continental cool summer | 1.28 | 1.08-1.50 | 1.27 | 1.07-1.51 | 5.23 (5) | 4.40 | 0.389 |
| Other | 1.25 | 1.07-1.46 | 1.32 | 0.85-2.04 | 15.20 (3) | 86.8 | 0.000 |
| ***Follow-up in years*** |  |  |  |  |  |  |  |
| >3 years | 1.22 | 1.06-1.41 | 1.25 | 0.89-1.76 | 21.81 (6) | 72.5 | 0.001 |
| ≤3 years | 1.28 | 1.10-1.50 | 1.26 | 0.97-1.63 | 10.11 (6) | 50.5 | 0.072 |
| ***Exposure assessment method*** |  |  |  |  |  |  |  |
| Home inspection | 1.16 | 0.83-1.63 | 1.45 | 0.78-2.67 | 21.20 (6) | 67.0 | 0.003 |
| Self-report | 1.26 | 1.13-1.41 | 1.25 | 1.01-1.55 | 10.70 (5) | 62.2 | 0.030 |
| ***Definition of asthma*** |  |  |  |  |  |  |  |
| Doctor-diagnosed/lung function measurements | 1.10 | 0.94-1.28 | 1.21 | 0.87-1.70 | 24.01 (8) | 66.7 | 0.002 |
| Self-report | 1.40 | 1.21-1.62 | 1.40 | 1.02-1.54 | 3.03 (4) | 0.80 | 0.388 |
| ***Quality*** |  |  |  |  |  |  |  |
| High (scores 8-9) | 1.31 | 1.51-1.49 | 1.25 | 1.00-1.56 | 15.00 (7) | 53.3 | 0.036 |
| Low (scores < 8) | 1.14 | 0.95-1.37 | 1.34 | 0.83-2.15 | 15.70 (5) | 74.5 | 0.003 |

**Legend**

^a^Large study: Cohort studies, n > 700; case-control studies, n > 181, where n= study size.
