# Supplementary material for: Residential Dampness and Molds and the Risk of Developing Asthma: A Systematic Review and Meta-Analysis
Source: PLoS One. 2012 Nov 7;7(11):e47526. doi: 10.1371/journal.pone.0047526 (PMC3492391; doi:10.1371/journal.pone.0047526)
Supplement: Table S8 — Summary effect estimates (EEs) for the relation between mold odor and the risk of asthma onset (n = 8) and stratified analysis according to the study characteristics. (DOCX) [file pone.0047526.s009.docx]

**Table S8.**  Summary effect estimates (EEs) for the relation between mold odor and the risk of asthma onset (n=8) and stratified analysis according to the study characteristics

| **Stratification** | **Model** | | | | **Heterogeneity Statistics** | | |
| --- | --- | --- | --- | --- | --- | --- | --- |
|  | **Fixed-effects model**  **EE (95%CI)** | | **Random-effects model**  **EE (95%CI)** | | **Q (n)** | **I^2^- statistics**  **(%)** | **P value** |
| **Main analysis** | 1.56 | 1.25-1.95 | 1.73 | 1.19-2.50 | 14.85 (8) | 52.9 | 0.038 |
| **Stratified analysis** |  |  |  |  |  |  |  |
| ***Study population*** |  |  |  |  |  |  |  |
| Infants (0 to 4 years) | 1.64 | 1.02-2.65 | 1.64 | 1.02-2.65 | 2.43 (4) | 0.0 | 0.488 |
| Children (up to 16 years) | 1.73 | 1.29-2.32 | 1.84 | 1.06-3.23 | 10.11 (4) | 70.3 | 0.018 |
| ***Study design*** |  |  |  |  |  |  |  |
| Cohort | 2.05 | 1.33-3.15 | 1.94 | 1.13-3.33 | 4.37 (3) | 31.1 | 0.224 |
| Incident case-control | 1.41 | 1.09-1.83 | 1.63 | 0.98-2.71 | 8.40 (5) | 64.3 | 0.038 |
| ***Study size*^a^** |  |  |  |  |  |  |  |
| Large | 1.61 | 1.27-2.03 | 1.91 | 1.24-2.95 | 13.47 (6) | 62.9 | 0.019 |
| Small | 1.12 | 0.53-2.38 | 1.12 | 0.53-2.38 | 0.58 (2) | 0.0 | 0.455 |
| ***Geographical location*** |  |  |  |  |  |  |  |
| USA | 0.99 | 0.64-1.54 | 0.99 | 0.64-1.54 | 0.66 (2) | 0.0 | 0.416 |
| Europe | 1.44 | 1.11-1.88 | 1.75 | 1.05-2.92 | 12.92 (6) | 61.3 | 0.024 |
| ***Climatic zone*** |  |  |  |  |  |  |  |
| Subarctic | 1.15 | 0.84-1.58 | 1.39 | 0.74-2.62 | 5.82 (4) | 48.4 | 0.121 |
| Continental cool summer | 1.49 | 1.08-2.07 | 1.59 | 1.01-2.81 | 8.63 (3) | 65.2 | 0.035 |
| ***Follow-up in years*** |  |  |  |  |  |  |  |
| >3 years | 1.77 | 1.32-2.35 | 1.91 | 1.15-3.19 | 10.55 (4) | 62.1 | 0.032 |
| ≤3 years | 1.14 | 0.85-1.53 | 1.20 | 0.82-1.76 | 3.88 (4) | 22.7 | 0.275 |
| ***Exposure assessment method*** |  |  |  |  |  |  |  |
| Home inspection | 1.64 | 1.02-2.65 | 1.64 | 1.02-2.65 | 2.43 (3) | 0.0 | 0.488 |
| Self-report | 1.54 | 1.19-1.98 | 1.86 | 1.06-3.26 | 12.36 (5) | 75.5 | 0.006 |
| ***Definition of asthma*** |  |  |  |  |  |  |  |
| Doctor-diagnosed/lung function measurements | 1.38 | 1.11-1.71 | 1.52 | 1.03-2.24 | 17.67 (7) | 60.4 | 0.014 |
| Self-report | - |  |  |  |  |  |  |
| ***Quality*** |  |  |  |  |  |  |  |
| High (scores 8-9) | 1.07 | 0.82-1.38 | 1.10 | 0.79-1.53 | 5.19 (4) | 23.0 | 0.268 |
| Low (scores < 8) | 2.28 | 1.64-3.18 | 2.28 | 1.64-3.18 | 0.97 (4) | 0.0 | 0.808 |

^a^Large study: Cohort studies, n > 700; case-control studies, n > 181, where n= study size.
