# Supplementary material for: Correction: Residential Dampness and Molds and the Risk of Developing Asthma: A Systematic Review and Meta-analysis
Source: PLoS One. 2014 Mar 26;9(3):e93454. doi: 10.1371/journal.pone.0093454 (PMC3966895; doi:10.1371/journal.pone.0093454)
Supplement: Table S3 — Effect estimates reported in the studies included in the meta-analysis (the lowest effect estimates reported in the studies). [file pone.0093454.s001.pdf]

The effect estimate of Gunnbjornsdottir MI [20] 2006 (1st column on the left) for any exposure (2nd column from the left) was incorrect in the original manuscript. The correct estimate is 1.13 (0.92-1.40).

Table S3. Effect estimates reported in the studies included in the meta-analysis (the lowest effect estimates reported in the studies)

**Reference, Year, Country**	**EE for exposure measures**				
	**Any exposure** EE (95%CI)	**Water damage** EE (95%CI)	**Dampness** EE (95%CI)	**Visible mold** EE (95%CI)	**Mold odor** EE (95%CI)
¥Nafstad P [13] 1998 Norway	3.8 (2.0-7.2)[b]				
Gent JF [14] 2002 USA	1.18 (0.90-1.55)[c]	1.18 (0.90-1.55)		1.23 (0.94-1.61) [a]	
¥Jaakkola MS [6] 2002 Finland	0.90 (0.61-1.34)[c]	0.90 (0.61-1.34)	1.02 (0.73-1.41)	0.98 (0.68-1.40)	0.98 (0.68-1.40)
McConnell R [15] 2002 USA	0.87 (0.68-1.12)[c]	1.08 (0.78-1.49)		0.87 (0.68-1.12)	
¥Rönmark E [16] 2002 Sweden	1.17 (0.58-2.40)[c]		1.17 (0.58-2.40)		
¥Belanger K [17] 2003 USA	1.54 (1.09-2.18) [c]			1.54 (1.09-2.18)	
¥Emenius G [18] 2004 Sweden	2.0 (1.20-3.40) [b]		1.50 (1.00-2.30)	1.00 (0.50-1.70)	2.00 (1.00-3.90)
¤Jaakkola JJK [19] 2005 Finland*	1.01 (0.66-1.54) [b]	1.01 (0.45-2.26)	0.92 (0.54-1.54)	0.65 (0.24-1.72)	2.44 (1.07-5.60)
¥Gunnbjornsdottir MI [20] 2006 (Iceland, Norway, Sweden, Denmark, Estonia)	1.13 (0.92-1.40) [b]	1.18 (0.95-1.44)	1.67 (1.22-2.27)	1.53 (1.18-1.98)	
¥Pekkanen J [21] 2007 Finland	1.97 (1.00-3.90)[c]		1.97 (1.00-3.90)	4.01 (1.12-14.32)	2.96 (0.62-14.19)
¥Karvonen AM [22] 2009 Finland	0.66 (0.14-3.17)[c]		1.29 (0.50-3.32)	5.22 (1.48-18.35)	0.66 (0.14-3.17)
¥Rosenbaum PF [23] 2010 USA β	0.90 (0.35-2.29)[c]	1.32 (0.58-3.22) [a]	1.32 (0.58-3.02) [a]	0.90 (0.35-2.29) [a]	1.32 (0.58-3.02) [a]
¥Schroer KT [24] 2009 USA	2.47 (1.27-4.80) [b]				
¥Hwang BF [25] 2011 Taiwan	1.69 (1.30-3.37) [b]	2.80 (0.59-13.3)		1.76 (1.18-2.26)	2.09 (1.30-3.37)
¥Larsson M [26] 2011 Sweden µ	0.93 (0.55-1.57) [c]	0.93 (0.55-1.57)	1.49 (0.35-6.30)	1.49 (0.35-6.30)	2.99 (1.50-5.94)
¥Reponen [27] 2011 USA	0.88 (0.52-1.48)[c]				

Legend

EE, effect estimate, either odds ratio or incidence rate ratio; Cl, confidence interval; µ, reported effect estimate for dampness and visible mold; *, reported effect estimate for visible mold and mold odor; β, reported effect estimate for dampness and mold odor; a, unadjusted OR; b, estimates for any exposure indicator reported in the studies; c, lowest effect estimates reported in the studies and used as any exposure indicator in the meta-analysis
