# Supplementary material for: Correction: Residential Dampness and Molds and the Risk of Developing Asthma: A Systematic Review and Meta-analysis
Source: PLoS One. 2014 Mar 26;9(3):e93454. doi: 10.1371/journal.pone.0093454 (PMC3966895; doi:10.1371/journal.pone.0093454)
Supplement: Table S4 — Summary effect estimates (EEs) for the relation between any exposure (including the highest effect estimates in the studies) and the risk of asthma onset (n = 16) and stratified analysis according to the study characteristics. [file pone.0093454.s002.pdf]

The summary effect estimates for any exposure (including the highest effect estimates) and the risk of asthma onset for both the fixed- and random- effects models and the heterogeneity statistics for the main analysis, studies on adults, cohort studies, large studies, European studies, studies from other climatic zones, studies with follow-up > 3 years, studies applying self-report for exposure assessment, studies applying self-report for outcome assessment and high quality studies were incorrect. The correct data is shown in Tables S4.

Table S4. Summary effect estimates (EEs) for the relation between any exposure (including the highest effect estimates in the studies) and the risk of asthma onset (n=16) and stratified analysis according to the study characteristics

Stratification	Model				Heterogeneity Statistics		
	Fixed-effects model EE (95%CI)		Random-effects model EE (95%CI)		Q (n)	I^2- statistics (%)	P value
**Main analysis**	1.32	1.20-1.46	1.48	1.23-1.78	38.75 (16)	61.3	0.001
**Stratified analysis**							
Study population							
Infants (0 to 4 years)	1.63	1.39-1.91	1.99	1.45-2.73	18.63 (8)	62.4	0.009
Children (up to 16 years)	1.23	1.02-1.48	1.30	1.01-2.02	10.43 (6)	52.1	0.064
Adults	1.10	0.92-1.31	1.10	0.92-1.31	0.26 (2)	0.0	0.607
Study design							
Cohort	1.27	1.20-1.46	1.34	1.11-1.63	21.26 (11)	53	0.019
Incident case-control	1.57	1.26-1.96	1.97	1.19-3.28	16.92 (5)	76.4	0.008
Study size[a]							
Large	1.35	1.18-1.34	1.64	1.24-2.18	29.54 (10)	69.5	0.001
Small	1.29	1.11-1.50	1.30	1.03-1.65	9.05 (6)	44.7	0.107
Geographical location							
USA	1.31	1.13-1.52	1.32	1.07-1.62	7.94 (6)	37.1	0.159
Europe	1.31	1.13-1.59	1.69	1.21-2.36	29.24 (8)	73.1	0.000
Climatic zone							
Subarctic	1.15	0.91-1.45	1.40	0.89-2.20	10.11 (5)	60.4	0.039
Continental cool summer	1.60	1.37-1.86	1.79	1.34-2.39	18.53 (8)	62.2	0.010
Other	1.16	0.99-1.37	1.18	0.97-1.44	2.70 (3)	26.1	0.29
Follow-up in years							
>3 years	1.20	1.04-1.39	1.32	1.01-1.72	14.24 (7)	57.9	0.027
≤3 years	1.46	1.27-1.68	1.66	1.26-1.80	23.02 (9)	65.2	0.003
Exposure assessment method							
Home inspection	2.22	1.67-2.94	2.24	1.47-3.41	11.92 (7)	49.6	0.089
Self-report	1.23	1.08-1.47	1.26	1.08-1.47	14.56 (9)	45.1	0.068
Definition of asthma							
Doctor-diagnosed/lung function measurements	1.31	1.12-1.53	1.57	1.15-2.13	31.34 (11)	68.1	0.001
Self-report	1.34	1.17-1.53	1.44	1.15-1.80	9.56 (5)	58.1	0.049
Quality							
High (scores 8-9)	1.22	1.08-1.37	1.22	1.02-1.44	11.30 (8)	38.1	0.126
Low (scores < 8)	1.62	1.35-1.95	1.99	1.38-2.88	22.83 (8)	69.3	0.002

Legend

[a]Large study: Cohort studies, n > 700; case-control studies, n > 181, where n= study size.
