# Supplementary material for: Correction: Residential Dampness and Molds and the Risk of Developing Asthma: A Systematic Review and Meta-analysis
Source: PLoS One. 2014 Mar 26;9(3):e93454. doi: 10.1371/journal.pone.0093454 (PMC3966895; doi:10.1371/journal.pone.0093454)
Supplement: Table S5 — Summary effect estimates (EEs) for the relation between any exposure (including the lowest effect estimates in the studies) and the risk of asthma onset (n = 16) and stratified analysis according to the study characteristics. [file pone.0093454.s003.pdf]

The summary effect estimates for any exposure (including the lowest effect estimates) and the risk of asthma onset for both the fixed- and random- effects models and the heterogeneity statistics for the main analysis, studies on adults, cohort studies, large studies, European studies, studies from other climatic zones, studies with follow-up > 3 years, studies applying self-report for exposure assessment, studies applying self-report for outcome assessment and high quality studies were incorrect. The correct data is shown in **Tables S5**

Table S5. Summary effect estimates (EEs) for the relation between any exposure (including the lowest effect estimates in the studies) and the risk of asthma onset (n=16) and stratified analysis according to the study characteristics

Stratification	Model				Heterogeneity Statistics		
	Fixed-effects model EE (95%CI)		Random-effects model EE (95%CI)		Q (n)	I^2- statistics (%)	P value
**Main analysis**	1.21	1.09-1.34	1.27	1.06-1.53	38.12 (16)	60.7	0.000
**Stratified analysis**							
Study population							
Infants (0-4 years)	1.54	1.31-1.81	1.69	1.26-2.27	17.38 (8)	59.7	0.015
Children (up to 16 years)	1.00	0.84-1.18	1.03	0.83-1.26	6.29 (6)	20.5	0.274
Adults	1.06	0.89-1.26	1.05	0.85-1.29	1.31 (2)	23.4	0.253
Study design							
Cohort	1.26	1.01-1.44	1.44	1.10-1.88	27.19 (5)	66.9	0.001
Incident case-control	1.59	1.27-2.00	1.80	1.12-2.90	18.27 (11)	45.3	0.051
Study size[a]							
Large	1.31	1.19-1.54	1.41	1.10-1.91	25.83 (10)	65.2	0.051
Small	1.13	0.98-1.30	1.10	0.85-1.42	11.17 (6)	55.2	0.048
Geographical location							
USA	1.17	1.02-1.35	1.21	0.91-1.62	15.77 (6)	68.3	0.008
Europe	1.26	1.10-1.44	1.44	1.10-1.88	27.19 (9)	66.9	0.001
Climatic zone							
Subarctic	1.12	0.71-1.43	1.30	0.79-2.01	10.11 (5)	60.4	0.059
Continental cool summer	1.60	1.37-1.86	1.79	1.34-2.39	18.53 (8)	62.2	0.010
Other	1.09	0.93-1.27	1.13	0.84-1.52	5.85 (3)	65.8	0.0.054
Follow-up in years							
>3 years	1.07	0.93-1.24	1.08	0.91-1.28	7.22 (7)	16.9	0.301
≤3 years	1.40	1.21-1.62	1.47	1.11-1.96	22.99 (9)	65.2	0.003
Exposure assessment method							
Home inspection	1.89	1.45-2.48	1.75	1.15-2.65	13.25 (7)	57.1	0.034
Self-report	1.14	1.02-1.27	1.13	0.97-1.32	14.09 (9)	43.2	0.079
Definition of asthma							
Doctor-diagnosed/lung function measurements	1.08	0.93-1.25	1.19	0.91-1.56	26.36 (11)	62.1	0.003
Self-report	1.32	1.16-1.51	1.43	1.13-1.80	1.10 (5)	60.0	0.0.041
Quality							
High (scores 8-9)	1.15	1.02-1.30	1.14	0.98-1.32	8.93 (8)	21.6	0.258
Low (scores < 8)	1.28	1.09-1.51	1.61	1.10-2.37	31.77 (8)	78.0	0.000

Legend

[a]Large study: Cohort studies, n > 700; case-control studies, n > 181, where n= study size.
